# Multi-state models for investigating possible stages leading to bipolar disorder

**DOI:** 10.1186/s40345-014-0019-4

**Published:** 2015-02-24

**Authors:** Charles DG Keown-Stoneman, Julie Horrocks, Gerarda A Darlington, Sarah Goodday, Paul Grof, Anne Duffy

**Affiliations:** 1 Department of Mathematics and Statistics, University of Guelph, 50 Stone Road East, Guelph, N1G 2W1 Canada; 2 Department of Epidemiology Dalla Lana School of Public Health, University of Toronto, 155 College St., Toronto, M5T 3M7, Canada; 3 Department of Psychiatry and Hotchkiss Brain Institute (HBI), University of Calgary, TRW Building, 3280 Hospital Drive, NW, Room 4D6, Calgary, AB, Calgary, T2N 4Z6 Canada; 4 Mood Disorders Centre of Ottawa, University of Ottawa Health Services, 1 Nicholas Street, Suite 302, Ottawa, K1N 7B7 Canada

**Keywords:** Bipolar disorder, Multi-state model, Survival analysis, High-risk

## Abstract

**Background:**

It has been proposed that bipolar disorder onsets in a predictable progressive sequence of clinical stages. However, there is some debate in regard to a statistical approach to test this hypothesis. The objective of this paper is to investigate two different analysis strategies to determine the best suited model to assess the longitudinal progression of clinical stages in the development of bipolar disorder.

**Methods:**

Data previously collected on 229 subjects at high risk of developing bipolar disorder were used for the statistical analysis. We investigate two statistical approaches for analyzing the relationship between the proposed stages of bipolar disorder: 1) the early stages are considered as time-varying covariates affecting the hazard of bipolar disorder in a Cox proportional hazards model, 2) the early stages are explicitly modelled as states in a non-parametric multi-state model.

**Results:**

We found from the Cox model thatthere was evidence that the hazard of bipolar disorder is increased by the onset of major depressive disorder. From the multi-state model, in high-risk offspring the probability of bipolar disorder by age 29 was estimated as 0.2321. Cumulative incidence functions representing the probability of bipolar disorder given major depressive disorder at or before age 18 were estimated using both approaches and found to be similar.

**Conclusions:**

Both the Cox model and multi-state model are useful approaches to the modelling of antecedent risk syndromes. They lead to similar cumulative incidence functions but otherwise each method offers a different advantage.

## Background

**Figure 1:**
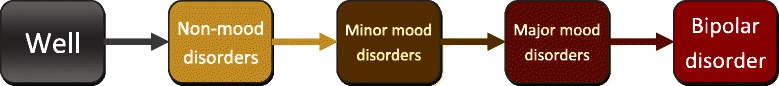
**Proposed stages for bipolar disorders.**

There is increasing evidence that non-mood and depressive disorders in children at familial risk reflect the early stages in the development of bipolar disorder (Duffy et al. 2010). Duffy et al. (2010) suggested that specific types of psychopathological manifestations are precursors forbipolar disorder in this high-risk population. Specifically, they showed that individuals at familial risk for bipolar disorder develop the illness in a forward sequence of clinical stages: evolving from non-mood disorders followed by minor mood disorders, then major mood disorders, and finally bipolar disorder (Figure 1).

In this developmental clinical staging model, non-mood disorders include primarily anxiety and sleep disorders, and in a subgroup of offspring from lithium non-responsive parents, attention deficit hyperactivity disorders (ADHD), and learning disabilities. Minor mood disorders comprise depressive disorders not otherwise specified, dysthymia, cyclothymia and adjustment disorders. Major mood disorder refers to major depressive disorder (single episode or recurrent). The last category, bipolar disorders, includes bipolar not otherwise specified, bipolar I and bipolar II disorders, and in the offspring of lithium non-responders schizoaffective disorder. Understanding the natural history of psychiatric disease is a critical advance supporting the identification of associated markers of illness risk and development. This approach has been very successful in other complex heritable medical diseases such as cancer and cardiovascular illness (Carlson et al. 2009; Thompson et al. 2007; Yancy et al. 2013). It is pertinent to test and develop the most rigorous analytic techniques to test the latter association as they involve the use of complex longitudinal data, and appropriate statistical analysis of data obtained from longitudinal investigations remains an important but challenging problem.

The Cox proportionalhazard model Cox (1972) is a common and familiar choice among investigators to examine longitudinal time to event data. However, there is concern over the validity of Cox models in the presence of time-varying covariates (Kalbfleisch and Prentice 2002). It has been suggested that multi-state models can be used as an alternative to Cox models to address some of these limitations (Cortese and Andersen 2010).

The clinical staging model proposed by Duffy et al. (2010) has previously been investigated using parametric multi-state models (Duffy et al. 2014). In order to assess the effectiveness of multi-state models when compared to Cox models we fit a non-parametric multi-state model with states representing the proposed stages of bipolar disorder and compared this to a Cox proportional hazards model in which the preceding stage of major depressive disorder is treated as a time-varying covariate (Cox 1972). The focus of this analysis is the effect of major depressive disorder on the risk of developing bipolar disorder in order to more easily demonstrate the statistical comparability between Cox models and multi-state models.

## Methods

### Data background

In compliance with the Helsinki Declaration, this research was approved by local research ethics boards in Ottawa, Halifax, and Calgary. As part of an ongoing longitudinal study, data were collected on 229 offspring from well characterized families with one parent having a confirmed history of bipolar disorder based on the best estimate procedure and blind consensus review (Duffy et al. 2007). In all families that were included, the other parent had no lifetime history of major affective disorder, schizophrenia, schizoaffective disorder, substance use disorder or personality disorder at baseline (Duffy et al. 2007). Bipolar disorder is highly heritable, therefore these offspring were at a much higher risk of developing bipolar disorder than the general public (Duffy et al. 2007; McGuffin et al. 2003). All offspring were recruited into the study between the ages of 7 and 25 from eligible families in which the proband parent was receiving long-term treatment in a mood disorders specialty clinic in either Ottawa or Halifax (Duffy et al. 2007). A more detailed description of the collection methods can be found in Duffy et al. (2007).

The high-risk subjects were re-evaluated annually to ascertain if they had developed any new psychopathology according to DSM-IV criteria (American Psychiatric Association 2000; Duffy et al. 2010). In addition to a research psychiatrist that conducted the interviews, all diagnoses were also confirmed by “blind consensus review using all available clinical information by at least two additional research psychiatrists” (Duffy et al. 2010). The total number of subjects that experienced each diagnosis is shown in Table 1.

**Table 1:** **Number of subjects that experienced the different diagnoses from the full dataset with 229 subjects**

**No Diagnoses**	**Non-mood**	**Minor Mood**	**Major Mood**	**Bipolar**
61	92	91	83	21

Whenever possible, multiple offspring were recruited from the same family. Therefore, of the 229 subjects, 99 individual families were represented. In order to preserve independence between observations, one subject from each of the 99 families was randomly selected for this analysis. The number of observed diagnoses from the randomly selected subjects is shown in Table 2. The dates of episodes were documented through prospective observation and diagnoses were decided on consensus review and based on semi-structured clinical interviews by research psychiatrists. For the analyses performed in this paper the time scale is age in years rather than calendar time.

**Table 2:** **Number of subjects that experienced the different diagnoses from the subset of the dataset with 99 subjects**

**No Diagnoses**	**Non-mood**	**Minor Mood**	**Major Mood**	**Bipolar**
21	45	40	38	10

### Cox models

Often when studying the probability of a single event occurring over time, survival analysis is used. An important concept in survival analysis is the hazard function, *α*(*t*). The hazard function is the rate at which an event occurs at time *t* given that the event has not occurred up to the instant before time *t*,

$$ \alpha (t)= \lim\limits_{\Delta t \to 0}\left(\frac{\mathbb{P}(T\leq t + \Delta t | T \geq t)}{\Delta t}\right), $$

where T is the event time and $\mathbb {P}(T\leq t)$ is the probability that the event has occurred before or at time *t*. When the effects of covariates are of interest, a commonly used method of analysis is the Cox proportional hazards model (Cox 1972).

The Cox model is a form of semi-parametric regression which allows covariates to affect the hazard of some event. In this case the hazard rate is modelled as:

(1) $$ \alpha \left(t|\textbf{x}_{i}(t)\right) = \alpha_{0} (t)e^{\beta_{1} x_{1i}(t) + \beta_{2} x_{2i}(t) + \cdots + \beta_{p} x_{pi}(t)},  $$

where *α*_0_(*t*) is a baseline hazard function that is often unspecified, *β*_1_,*β*_2_,…,*β*_*p*_ are regression coefficients, and *x*_1*i*_(*t*),*x*_2*i*_(*t*),…,*x*_*pi*_(*t*) are covariates for subject *i*. Note that the covariates can change with time. We can interpret the effect of a covariate as follows: A one unit increase in *x*_*i*1_(*t*) is estimated to multiply the hazard by $e^{\beta _{1}}\phantom {\dot {i}\!}$, holding all other covariates constant.

Another function of interest is the cumulative incidence, the probability of experiencing the event before time *t* (Breslow 1972; Klein and Moeschberger 2003):

(2) $$ \begin{aligned} CIF(t) &= \mathbb{P}\left(T\le t\right) \\ &=1-e^{-{\int_{0}^{t}}\alpha(u)du} \\ &=1-e^{-A(t)}, \end{aligned}  $$

where A(t) is the cumulative hazard. In practice, events are observed at discrete times, *t*_(1)_≤*t*_(2)_≤…≤*t*_(*n*)_ and the cumulative incidence can be estimated using the Breslow estimator (Breslow 1972):

(3) $$ \widehat{CIF}(t)=1-\exp\left\{ -\sum_{s\le t} \Delta \hat{A}(s) \right\},  $$

where the sum is over all event times observed up to time *t*, and $\Delta \hat {A}(s)$ is the change in the estimated cumulative hazard at time *s*. Suppose we want to estimate the CIF for a person with a single time-varying covariate that takes the value *x*^∗^(*s*) at time *s*. The estimator used by the survfit function (Therneau 2000) R (R Core Team 2013) can be expressed as (modified from (Kalbfleisch and Prentice 2002)):

(4) $$ \Delta \hat{A}(s)=\frac{\delta (s)\exp\left\{ \hat{\beta} x^{*}(s)\right\}}{\sum_{i=1}^{n} Y_{i}(s)\exp\left\{ \hat{\beta} x_{i}(s)\right\}}  $$

where *δ*(*s*) is the number of events at time *s*, *Y*_*i*_(*s*) is an indicator variable which is 1 if subject *i* is at risk at time *s*, and *x*_*i*_(*s*) is the value of the covariate for subject *i* at time *s*.

Kalbfleisch and Prentice (2002) identified the importance of distinguishing between internal and external covariates when using a Cox model. External covariates are completely determined at the start of the study (e.g. a person’s sex or age) or are random but external to the subject (e.g. the weather). Internal covariates are random measurements on the subject that vary with time and may be affected if the event of interest occurs. For example, if the event of interest is death, then blood pressure would be an internal time-varying covariate, as it is a characteristic of the subject that varies with time and is affected by the event of interest, in the sense that it cannot be measured after death. Modelling of internal covariates can be difficult as the effect of the covariate on the event of interest may be complicated by the effect of time. For example, the effect of a covariate may not be observed until a time lag has passed (Kalbfleisch and Prentice 2002). Assuming that the effect of the internal covariate is correctly specified, including the correct lag and functional form, the interpretation of a regression coefficient in (1) is still valid (Kalbfleisch and Prentice 2002). However, estimates of the cumulative incidence function corresponding to (2) are considered invalid in the presence of internal covariates because the distribution of the covariate may depend on the event time (Kalbfleisch and Prentice 2002). Multi-state models are sometimes suggested as a solution to this problem (Cortese and Andersen 2010).

In this paper, we will consider Cox models with time to bipolar disorder as the response, and preceding diagnoses as time-varying covariates. Preceding diagnoses are considered internal covariates because they are measured on the subject and vary with time, and because once a diagnosis is received, the subject cannot subsequently receive a diagnosis of a less severe disorder, in accordance with diagnostic convention (American Psychiatric Association 2000). However, there is a problem with this approach, namely that traditional survival analysis allows only one outcome. On the other hand, we are interested in building a more complicated model. Therefore, in the next section we discuss multi-state models that allow multiple outcomes.

In this paper, preceding diagnoses are coded so that they take a value of 0 before and 1 after the diagnosis. The Cox models were fit using the coxph function from the survival R package using R version 3.0.2 for Windows 64bit (R Core Team 2013).

### Multi-state model

In multi-state models, individuals move between states over time. The parameters of interest are the transition rates between states, also called the intensities. An important assumption often used to simplify multi-state models is the Markov assumption, which assumes that the transition rate is independent of both the length of stay in the current state (sojourn time) and which states were visited prior to the current state. For a Markov multi-state model, the rate of transitioning from state *h* to another state *j* at time *t* is given by (Andersen and Keiding 2002):

(5) $$ \lambda_{hj}(t)=\lim\limits_{\Delta t \to 0}\left(\frac{P_{hj}\left(t,t+\Delta t\right)}{\Delta t}\right),  $$

where *P*_*hj*_(*s*,*t*) is the probability of being in state *j* at time *t* given a subject was in state *h* at time *s*.

The state probabilities can be expressed in matrix form as



with entries *P*_*hj*_(*s*,*t*), where *I* is the identity matrix,  is a product integral (Kalbfleisch and Prentice 2002), and *Δ****Λ***(*u*) is a matrix with elements *ΔΛ*_*hj*_(*u*), the change in the cumulative transition rate between state *h* and state *j* at time *u*. State probabilities can be estimated using the Aalen-Johansen estimator $\widehat {\textbf {P}}\left (s,t\right)$, which is a matrix with elements $\widehat {P}_{\textit {hj}}(s,t)$ (Aalen and Johansen 1978),

(6) $$  \widehat{\textbf{P}}\left(s,t\right) = \prod_{u\in (s,t]}^{}\left(I + \Delta \widehat{\boldsymbol{\Lambda}}(u) \right),  $$

where $\Delta \widehat {\boldsymbol {\Lambda }}(u)$ is the change in the matrix $\widehat {\boldsymbol {\Lambda }}(u)$, which is a matrix with elements $\widehat {\Lambda }_{\textit {hj}}(u)$ which are estimates of the cumulative hazard of transitioning from state *h* to state *j* at time *u*,

$$\widehat{\Lambda}_{hj}(t) = \sum_{s\le t} \frac{\delta_{hj}(s)}{Y_{h}(s)}, $$

where *δ*_*hj*_(*s*) is the number of observed transitions from state *h* to state *j* at time *s*, and *Y*_*h*_(*s*) is the number of uncensored subjects in state *h* at time *s*. For multi-state models where all subjects start in state W at time 0, we can define the CIF for an absorbing state Z as:

(7) $$ \begin{aligned} CIF_{Z}(t)&=P(T_{Z} \le t) \\ &=P_{WZ}(0,t) \\ &= \left[ \prod_{u\in (0,t]}^{}\left(I + \Delta \boldsymbol{\Lambda}\left(u\right) \right) \right]_{W,Z} \end{aligned}   $$

where *T*_*Z*_ is the time of transition into state *Z* from any other state.

It has previously been noted that the choice of a state structure for a multi-state model is important, and not unique for each situation (Hougaard 1999; Keown-Stoneman 2013). A good state structure can simplify calculations and make interpretation of the model more straightforward. The state structure must be complicated enough to accommodate all the pathways observed in the data, yet simple enough to allow meaningful inference.

The mstate package for R was used to fit all multi-state models for this paper (de Wreede et al. 2010; R Core Team 2013). Using the mstate package, one can estimate cumulative transition rates ***Λ***(*u*), and state probabilities **P**(*s*,*t*).

## Results and discussion

### Cox model

A Cox model is now presented with time to bipolar disorder as the event of interest and one time-varying covariate representing the absence or presence of a major depressive disorder:

$$\alpha_{i}(t)=\alpha_{0}(t)\exp\{{\beta}_{1}x_{i}(t)\} $$

where *α*_*i*_(*t*) is the hazard of bipolar disorder for subject *i*, *i*=1,2,…,*n*; *α*_0_(*t*) is the baseline hazard, *β*_1_ is a regression coefficients to be estimated, and *x*_*i*_(*t*) is a time-varying covariate that is equal to 1 if subject *i* had major depressive disorder by time *t*, and 0 otherwise. Table 3 shows the results of the Cox model with major mood as a time-varying covariate.

**Table 3:** **Results from the Cox model**

**Covariate**	**Parameter**	**Standard**	***Z***	**p-value**	**Hazard**
	**estimate**	**error**			**ratio**
Major mood	1.6233	0.7147	2.271	0.0231	5.070

From the Cox model, there is evidence that the hazard of bipolar disorder is increased by the onset of major depressive disorder (p = 0.0231). In particular, it is estimated that having a diagnosis of major depressive disorder multiplies the hazard of bipolar disorder by 5.070 (Table 3).

### Multi-state model

**Figure 2:**
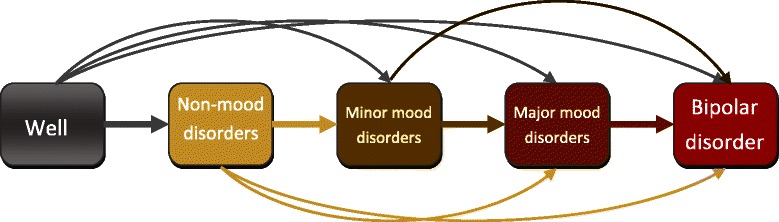
**State structure of 5-state multi-state model for bipolar disorder.**

As an alternative approach, we fit a multi-state model which has states representing the 5 different stages proposed in (Duffy et al. 2010). We defined an individual’s state at time *t* as the most severe diagnosis, up to that point. While the staging hypothesis illustrated in Figure 1 shows a linear progression through stages, not all subjects were observed to pass through every stage/state. To accommodate these individuals, skipping of stages is allowed in the multi-state model. However, backward transitions are not allowed. For instance, a person can move from well to major mood disorder (skipping non-mood disorder and minor mood disorder) but cannot transition from major mood disorder to minor mood disorder. The state structure of the 5-state multi-state model is shown in Figure 2.

**Figure 3:**
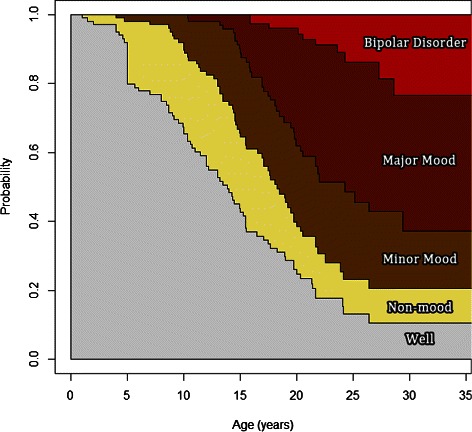
**Estimated probability of being in each state by age, based on the multi-state model.**

No regression estimates are available because the events that were previously treated as covariates are now treated as states. However, one method of visualizing the results of the multi-state model is shown in Figure 3. The horizontal axis represents age in years. The vertical axis shows the cumulative probability of being in a particular state or a less severe state. For instance, at age 20, the estimated probability of being in Non-mood or Well is approximately 0.40, and the probability of being in the Minor Mood, Major Depressive or Bipolar Disorder stage is approximately 1−0.40=0.60. The height of a shaded region at a particular age represents the estimated probability of being in the corresponding state at that age. Thus the probability of being in the Major Depressive stage at age 20 is approximately 0.61−0.40=0.21. Figure 3 was produced by the mstate package using the Aalen-Johansen estimator (de Wreede et al. 2010).

**Figure 4:**
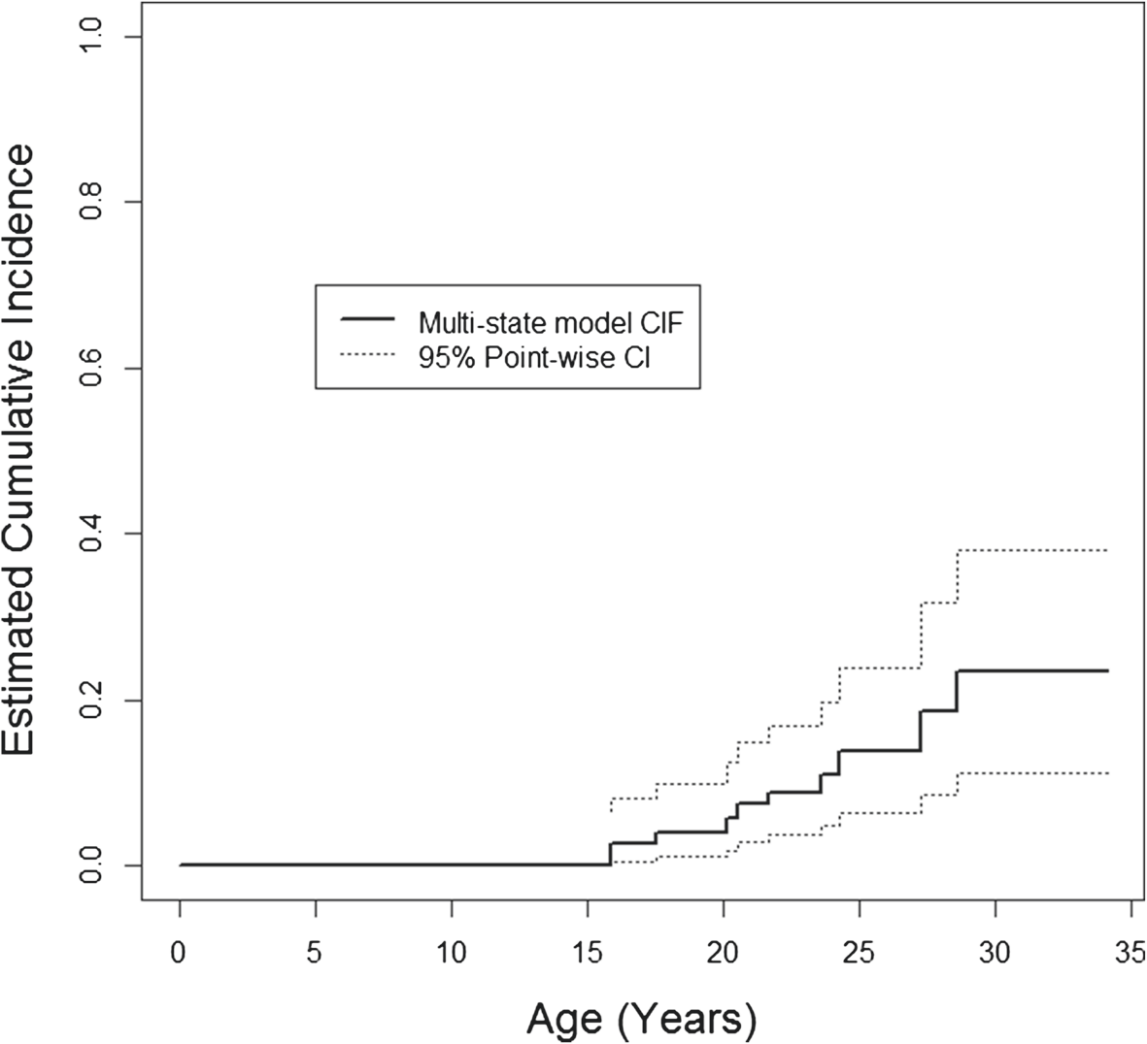
**Estimated cumulative incidence of bipolar disorder using the multi-state model.**

An estimate of the cumulative incidence of bipolar disorder can be obtained as in equation (7) with *Z*=*BD*. It was estimated that 23.21% of high-risk offspring developed bipolar disorder by age 29. This is shown in Figure 4. Ninety-five percent confidence intervals for the multi-state model CIF were obtained using the delta method to obtain point-wise standard errors and the complementary log-log approach to obtain point-wise confidence intervals (Kalbfleisch and Prentice 2002).

## Conclusions

### Comparison of models

When assessing a temporal association between two variables (e.g. depression and subsequent bipolar disorder), it is unclear how two popular methods, Cox proportional hazards and multi-state models compare. Both the Cox proportional hazards model and the multi-state model have benefits in the analysis of bipolar disorder. One advantage of treating previous events as states in a multi-state model approach over treating them as covariates in a Cox model is that the multi-state model does not assume proportional hazards. However, the Cox model has a more straightforward interpretation of covariate effects than non-parametric multi-state models and allows tests to be performed to determine if the effect of a covariate is significantly different from zero. A disadvantage of the multi-state model approach is that it cannot handle continuous internal covariates as states unless they are first transformed into categories, as they must be represented by discrete states. Also, since the hazard function is estimated separately for each transition, prediction based on non-parametric multi-state models may be more susceptible to bias due to sparse data.

As stated above, the focus of the Cox model is usually regression coefficients, while the focus of a multi-state model is state (stage) probabilities, hence the two approaches are generally not directly comparable. However, they will both produce conditional cumulative incidence functions (CIF). Here we estimated the conditional CIF of bipolar disorder after age 18 given a high-risk subject had major depressive disorder prior to age 18. The selection of major depressive disorder and 18 years old was arbitrarily chosen as an example of a conditional CIF. Since we assume the Markov property that the hazard of transitioning is independent of the length of stay in the current state, and that the subject has not been diagnosed with bipolar disorder before age 18, it is inconsequential when major depressive disorder occurred, as long as it was before age 18.

To estimate the conditional CIF given major mood was diagnosed prior to age 18, from the Cox model, we used the Breslow method to estimate the cumulative incidence (Breslow 1972; Klein and Moeschberger 2003):

$$\begin{array}{*{20}l} \widehat{CIF}^{(Cox)}\!\left(t|x(t)\,=\,1,t\ge 18\right)\! &= \!1\!- \!\exp\{\!-\hat{A}(t|x(t)\,=\,1,t\!\ge\! 18)\! \} \\ &=\! 1\!- \!\exp\!\left\{\,-\,\!\sum_{s=18}^{t}\!\Delta\hat{A}\!(s|x(s)\,=\,1\!)\! \right\}\!, \end{array} $$

where $\hat {A}(t|x(t)=1,t\ge 18)$ is the estimated cumulative hazard of bipolar disorder from time *t* onward, given major mood was diagnosed prior to age 18. This was estimated from a Cox model fit to the entire dataset, then the estimated changes in the cumulative hazard were used to construct the probability of bipolar disorder beyond age 18 given major mood was present.

To estimate the conditional CIF from the multi-state model, the Aalen-Johansen estimator of the transition probability matrix was used (Aalen and Johansen 1978). The estimated conditional CIF is then given by:

$$\begin{aligned} \widehat{CIF}^{(MSM)}\left(t|X(18)=MM\right)&=\widehat{\textbf{P}}\left(18,t\right)_{MM,BD} \\ &= \left[ \prod_{s=18}^{t}\left(I + \Delta \hat{\boldsymbol{\Lambda}}\left(s\right) \right) \right]_{MM,BD} \end{aligned} $$

where X(t) is the state occupied at time t, $\widehat {\textbf {P}}\left (18,t\right)$ is a matrix of the Aalen-Johansen estimates and $\widehat {\textbf {P}}\left (18,t\right)_{MM,BD}$ is the element that corresponds to the estimated probability of being in the bipolar disorder (*BD*) state at time *t* given the subject was in the major mood disorder (*MM*) state at time 18, *I* is the identity matrix, $\Delta \hat {\boldsymbol {\Lambda }}\left (s\right)$ is a matrix of the changes in the cumulative transition intensities at time *s*, and *s* indexes the observed transition times between states.

**Figure 5:**
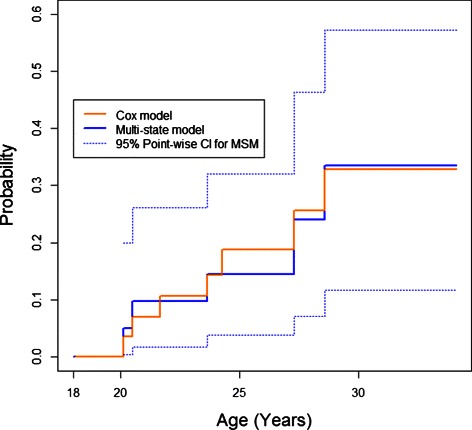
**Estimated conditional cumulative incidence of bipolar disorder after age 18 given major mood before age 18.**

Figure 5 shows the estimated probability of bipolar disorder given a diagnosis of major mood before age 18. It is apparent that the CIF from the Cox model has more steps than that from the multi-state model. This is because the Cox estimate jumps at every bipolar disorder event, while the multi-state model estimate only jumps at bipolar disorder events corresponding to an individual in the major depressive state. Nevertheless, it is clear that the two approaches produced very similar results. Furthermore, simulations (not shown) indicate that for exponentially distributed sojourn times, as sample size increases the two estimators converge.

Using the multi-state model approach we obtained estimates of the probability of high-risk offspring being in the various proposed stages of bipolar disorder. As an example analysis, when major mood disorder was assumed to have occurred prior to age 18, the Cox model estimate of the probability of bipolar disorder appears to be very similar to the multi-state model estimate (Figure 5). Using the same methods as in Figure 4, 95% confidence intervals were obtained for the multi-state model CIF in Figure 5 (Kalbfleisch and Prentice 2002).

In summary, if assessing the association between longitudinal variables is the primary interest for researchers then Cox models with time-varying covariates are recommended. If predicting the probability of entering future states, as in CIFs, is the primary interest then multi-state models are preferred. However, we have shown that Cox models can also be used to construct conditional CIFs. As shown in Figure 3 it is possible to include all of the stages of bipolar disorder as states in a multi-state model. Although this paper only included a Cox model with major mood disorder as a time-varying covariate for simplicity, it is also possible to include time-varying covariates that represent the presence of the two other previous stages, non-mood and minor mood. It would also be possible to test for interactions between the different stages.

It should be noted that multi-state models can also allow for other covariates to affect the transition intensities between states. Although the choice of using one randomly selected offspring from each family did not have a considerable effect on the results (data not shown) further work into the modelling of bipolar disorder could include more complex analyses that account for familial correlation.

Although they are unlikely to completely replace the Cox model, the multi-state model is another useful tool for investigating the relationships among different events over time.
